# A Review of Scrub Typhus (*Orientia tsutsugamushi* and Related Organisms): Then, Now, and Tomorrow

**DOI:** 10.3390/tropicalmed3010008

**Published:** 2018-01-17

**Authors:** Alison Luce-Fedrow, Marcie L. Lehman, Daryl J. Kelly, Kristin Mullins, Alice N. Maina, Richard L. Stewart, Hong Ge, Heidi St. John, Ju Jiang, Allen L. Richards

**Affiliations:** 1 Department of Biology, Shippensburg University, Shippensburg, PA 17202, USA; alfedrow@ship.edu (A.L.-F.); mllehm@ship.edu (M.L.L.); rlstew@ship.edu (R.L.S.); 2 Viral and Rickettsial Diseases Department, Naval Medical Research Center, Silver Spring, MD 20910, USA; kelly.350@osu.edu (D.J.K.); kmullins@som.umaryland.edu (K.M.); alice.n.maina.ctr@mail.mil (A.N.M.); hong.ge.ctr@mail.mil (H.G.); heidi.k.stjohn.ctr@mail.mil (H.S.J.); ju.jiang2.ctr@mail.mil (J.J.); 3 Department of Evolution, Ecology and Organismal Biology, The Ohio State University, Columbus, OH 43210, USA; 4 Department of Preventive Medicine and Biostatistics, Uniformed Services University of the Health Sciences, Bethesda, MD 20814, USA

**Keywords:** *Orientia tsutsugamushi*, *Orientia*, scrub typhus, mites, chiggers, rickettsia

## Abstract

Scrub typhus and the rickettsial diseases represent some of the oldest recognized vector-transmitted diseases, fraught with a rich historical aspect, particularly as applied to military/wartime situations. The vectors of *Orientia tsutsugamushi* were once thought to be confined to an area designated as the Tsutsugamushi Triangle. However, recent reports of scrub typhus caused by *Orientia* species other than *O. tsutsugamushi* well beyond the limits of the Tsutsugamushi Triangle have triggered concerns about the worldwide presence of scrub typhus. It is not known whether the vectors of *O. tsutsugamushi* will be the same for the new *Orientia* species, and this should be a consideration during outbreak/surveillance investigations. Additionally, concerns surrounding the antibiotic resistance of *O. tsutsugamushi* have led to considerations for the amendment of treatment protocols, and the need for enhanced public health awareness in both the civilian and medical professional communities. In this review, we discuss the history, outbreaks, antibiotic resistance, and burgeoning genomic advances associated with one of the world’s oldest recognized vector-borne pathogens, *O. tsutsugamushi.*

## 1. Introduction

*Orientia tsutsugamushi* is an obligate, intracellular bacterium and the causative agent of scrub typhus disease in humans. The World Health Organization has dubbed scrub typhus one of the world’s most underdiagnosed/underreported diseases that often requires hospitalization [1], highlighting the necessity for a better understanding of the vectors, outbreaks, and pathogenesis associated with this potentially fatal organism that has been linked to human cases/outbreaks both within and beyond its previously recognized region of endemicity. The genus *Orientia* belongs to the order Rickettsiales within the family Rickettsiaceae. In addition to scrub typhus caused by *O. tsutsugamushi* and newly identified *Orientia* species (*Candidatus* Orientia chuto and others) [2,3], the rickettsial (spotted fever and typhus group), ehrlichial, and anaplasmal diseases are also included within the order Rickettsiales [4]. 

The agents of the order Rickettsiales (both of known and unknown pathogenicity) are potentially transmitted to humans via the biting and/or contamination of mucous membranes/lesions by ectoparasites/arthropods such as hard or soft ticks, fleas, mosquitoes, mites, lice, and fleas [5,6,7,8,9]. Specifically, *O. tsutsugamushi* is vectored by the biting of the larval life stage of infected *Leptotrombidium* mites. The lifecycle of mites in the family Trombiculidae (Acari: Trombidiformes) consists of an egg, two six-legged stages: prelarva and larva, and four eight-legged stages: protonymph, deutonymph, tritonymph, and adult. The prelarval, proto- and tritonymphal stages are inactive and nonfeeding, while the deuteronymph and adult stages are non-parasitic and generally feed on arthropod eggs or small arthropods [10,11]. It is accepted that adults are the overwintering stage; however, several of these quiescent instars [12] may also open the ecological potential to persist during severe environmental conditions including overwintering [13]. Larval mites, often called chiggers, are the only parasitic stage. They use hair follicles and pores to gain entry into the skin and feed for 3–5 days using a stylostome (feeding tube) to inject salivary secretions that lyse host tissue [14]. This dissolved tissue is ingested by larval mites. Larval trombiculid mites attack every major group of terrestrial vertebrates, but mites in the genera known to vector *O. tsutsagamushi* primarily feed on small mammals [15]. In one study of hosts and larval mites in Yunnan Province, China, researchers collected 10,222 individual small mammals (representing 62 species) that were parasitized by 92,990 larval mites representing 224 species [16]; other studies have demonstrated a similar non-specific pattern of feeding on mammals [17,18,19]. Clearly, there is little host specificity, and larval mites feed opportunistically on a variety of mammals. 

While it was previously thought by some researchers that vertebrate hosts, especially mammals, may serve as reservoirs [20,21], it is now accepted that trombiculid mites are both the vector and reservoir for *O. tsutsugamushi* [22,23]. *O. tsutsugamushi* is maintained in trombiculid mites through transstadial and transovarial transmission. Transstadial transmission occurs when a pathogen is preserved through instars, i.e., *O. tsutsugamushi* is retained from the larval stage, through the protonymph and into the deutonymph stages. This can, with some pathogens and parasites, result in horizontal transmission when one stage (i.e., larva) acquires the infectious agent, and a later stage (i.e., deutonymph) infects a naive host. However, since trombiculid deutonymphs are not parasitic, it is assumed that horizontal transmission is not possible, though this does not preclude the situation for horizontal transmission during the phenomenon of pooled feeding (multiple chiggers feeding in close proximity on a rodent). Transovarial transmission (also known as vertical transmission) is the process by which the female passes the pathogen to the eggs. Transovarial infection rates can be 100% and transstadial passage is similarly high [24], permitting maintenance of *O. tsutsugamushi* in the mites. To re-infect the mite population with *O. tsutsugamushi*, larvae can also acquire the bacterium from mammals and while co-feeding on a naïve host with infected larvae [22,25]. *Leptotrombidium* sp. [15] have been demonstrated to be extremely competent vectors and reservoirs; however, another mite species, *Schoengastiella ligula*, in the same superfamily Trombiculoidea, but within the family Walchiidae, has also been implicated as a vector [26]. Since mites in the family Trombiculidae and the genus *Leptotrombidium* sp., as well as many other species, are established throughout the Western Hemisphere [27,28,29], the potential for establishment of *O. tsutsugamushi* or other *Orientia* species outside of the Tsutsugamushi Triangle is high. Recent reports of scrub typhus in Chile [3,30] and Peru [31] indicate that scrub typhus is endemic to South America. Though a vector for the *Orientia* species has not been identified, for one particular case on Chiloé Island, Chile, the patient remembered being bitten by a terrestrial leech [3]. The authors point out that terrestrial leeches do vector *Rickettsia* sp. [3,32,33]; however, several leeches from the area of the patient’s leech bite were negative for *Orientia* spp. (unpublished data). It is therefore still unknown what the vector(s) may be of the *Orientia* spp. of this and other patients in South America. Much more research needs to be conducted to investigate the emergence of *Orientia* species and their vector(s)/host(s) in the Western Hemisphere. 

*O. tsutsugamushi* possesses a small genome that has evolved in close association with arthropod hosts; therefore, *O. tsutsugamushi* cells are adapted to survive within the host cells [34]. This obligate, intracellular lifestyle precludes *Orientia* spp. from being cultivated on agar plates or in broth; instead, the organism is most commonly cultured in eukaryotic host cells within the required confines of biosafety level 3 (BSL3) laboratories. *O. tsutsugamushi* commonly infects endothelial cells, macrophages and polymorphonuclear leukocytes in patients and/or susceptible animals [35,36,37,38,39]. It can also grow in the yolk sac of 5–7 days-old embryonated chicken eggs, as well as established cell lines such as L929, Vero, BHK, McCoy and HeLa cells [40]. In addition, *O. tsutsugamushi* may grow in conventional hemocultures for a short time, which can be a useful technique for diagnostic purposes [41]. L929 cells tend to be the most commonly used host cells for the propagation of *O. tsutsugamushi* [42]. Irradiated L929 cells are grown as monolayers on the surface of culture flasks, and upon reaching 80% confluency, the cells are infected with a predetermined dilution of *O. tsutsugamushi* inoculum and subjected to a 1 h absorption period at room temperature, followed by incubation at 35 °C with 5% CO_2_ using maintenance media (5% FBS + RPMI 1640). After the *O. tsutsugamushi* cells initially enter into the host cells by phagocytosis, they are then released into the cytoplasm by disruption of the phagosomal membrane [43]. In our experience, the number of oriential cells increases (binary fission) day by day, with a doubling time of *O. tsutsugamushi* of approximately 8–9 h, until the host cell cytoplasm is filled with the orientiae at approximately 96 to 120 h post infection; however, this can be dependent upon the particular strain of *O. tsutsugamushi* being propagated, and reports have suggested that the median time for the detection of orientiae could be 27 days [44]. *O. tsutsugamushi* most commonly replicates in the host cell cytoplasm, but intranuclear localization has been observed in a few cases [45]. The oriential cells then invade neighboring cells through the process of budding [40,43]. 

Monitoring of the growth of *O. tsutsugamushi* can be visualized using quick stains with chemical reagents (e.g., Giemsa, Diff-Quik) and/or fluorescent dyes (e.g., acridine orange, live-dead stains) [46,47]. Recently, the use of differential fluorescent dyes to label distinct compartments of living *O. tsutsugamushi* cells has been described, including specific labeling of the cytoplasm, nucleic acids, cell membrane, and the cell wall of living and/or fixed *O. tsutsugamushi* [48]. These techniques enhance the ability to track living orientiae, and will thereby enable the study of processes such as attachment and entry of orientiae into host cells, as well as the intracellular trafficking and division within host cells. Moreover, these newer methods will also contribute to providing details concerning the fundamental processes in bacterial cell biology such as growth and elongation, and the generation of outer membrane vesicles [48]. In addition, the ImageJ software, a Java-based open-source image enumeration program, has been applied in the accounting of *O. tsutsugamushi* cultured in vitro [49]. This program demonstrates the highest correlation (r = 0.984) compared with the manual counting methods. Despite enhancements in the methods used to propagate and visualize orientiae in cell culture systems, the time factor required for growth, the necessity of skilled personnel with experience handling/manipulating rickettsiae/orientiae, and the requirement to use BSL3 facilities continue to be challenges faced by researchers in the past, present and, undoubtedly, the future.

The use of animal models has been incorporated into the laboratory growth/propagation of *O. tsutsugamushi*, as well as for the improved detailing of the pathogenesis associated with *Orientia* species. A variety of animals have long been employed for the study of *O. tsutsugamushi*; among which are non-human primates [50,51], guinea pigs [52], cotton rats [53], a variety of murine species (both inbred and outbred) [54,55,56,57,58], rabbits, gerbils and hamsters [59,60]. In particular, murine susceptibility to *O. tsutsugamushi* has been shown to be dependent upon the strain of *O. tsutsugamushi*, the mouse strain, and the route of infection/challenge method used. Early studies demonstrated that the intraperitoneal (IP) route of infection could induce illness/death in an array of inbred, outbred, and crossbred strains of mice [57]. These studies demonstrated that the Karp, Kato and Gilliam strains of *O. tsutsugamushi* proved to be some of the most lethal in all murine strains tested via the IP route of challenge. Beyond studies utilizing the IP route of challenge, researchers have utilized intradermal [61,62], subcutaneous [63], intravenous [64], intracranial [65] and intramuscular [65] murine challenge models in order to better discern the pathogenesis of *O. tsutsugamushi*. Similar to the IP route of challenge, the outcome of each type of challenge route is dependent upon the strain of *O. tsutsugamushi* being used and the background of the mice being employed in the study. Despite the recognized pathogenic differences associated with the use of various mouse challenge models, the goal of developing a model that reliably recapitulates the features of human scrub typhus infections remains a common goal of rickettsiologists aiming to further the vaccine/diagnostic efforts surrounding *O. tsutsugamushi.* Recently, intradermal [61,62], intravenous [66], and subcutaneous [67] challenge models, as well as mite-feeding models [68], have all been utilized in *O. tsutsugamushi* challenge/vaccine-challenge studies. Despite advances in the standardization of dosing strategies, and the increased sensitivity of detection via molecular/immunological techniques, differential outcomes (including target cell populations, bacterial loads, and immune response) have been observed in each model. For instance, IP challenge results in severe peritonitis that is often argued not to reflect the natural course of human infection; however, it is very reliable as a control for lethal models of infection. Arguments for the use of intravenous models of infection in inbred mice reason that intravenous infection mimics the pathology of fatal human scrub typhus cases; however, intravenous infection does not replicate the natural mode of transmission of infection by chigger to human. Intradermal models of infection mimic the natural mode of transmission, but often display slower/delayed kinetics of bacterial trafficking and onset of outward clinical symptoms. Consequently, the efforts to develop a murine model of *O. tsutsugamushi* that is broadly applicable to human scrub typhus cases have long been confounded by bacterial strain, murine strain and challenge route differences. In spite of these differences, the research aimed at establishing murine models of infection, particularly for the use in vaccine-challenge studies and identification of immune correlates of protection, continues to be of utmost importance to the characterization of cellular and molecular factors associated with the pathogenesis of the various strains (and potential new species) of *Orientia.*

Human diseases caused by agents within the order Rickettsiales can range from mild (asymptomatic) to lethal, and are generally flu-like (fever, headache, myalgia) in their symptomology; during severe infections, complications such as meningitis, intravascular complications, severe pneumonitis/peritonitis, and/or cardiac distress have been reported [69,70]. Moreover, a hemophagocytosis syndrome (HPS) can be associated with scrub typhus. The diagnosis of HPS can be made on cytologic findings of histiocytes containing phagocytosed blood cells in bone marrow aspirates. It is important to relate HPS with scrub typhus, as the prognosis if untreated can be poor [71,72,73]. It is noteworthy that both the spotted fever group rickettsioses and scrub typhus can include an inoculation eschar at the bite site, adding an additional challenge to differential diagnosis. The drug of choice for the treatment of diseases within the order Rickettsiales is doxycycline (tetracycline, chloramphenicol, and azithromycin have also been used successfully) [74,75,76,77]; however, the emergence of antibiotic-resistant strains of *O. tsutsugamushi* is of current/future concern [78]. Additionally, there are no long-lasting, broadly-protective vaccines available against scrub typhus (or the rickettsial diseases), despite a long, rich history of research attempts (killed, live, attenuated, subunit vaccines) aimed at the successful development of such [79]. It is known that *O. tsutsugamushi* is responsible for approximately one million cases of scrub typhus each year within endemic areas, and that an estimated one billion people per year are at risk of becoming infected [80]. In addition to the local residents within these endemic and higher risk areas, travelers/tourists, military personnel and their families, and government employees from around the globe can be found. Consequently, the emergence of new *Orientia* species, the increase in outbreaks both within and outside of the Tsutsugamushi Triangle, the emergence of antibiotic resistant strains of *Orientia*, and the sheer number of people at risk of contracting the disease, all underscore the vast importance of continued research efforts concerning the distribution, diagnostic development and vaccine efforts geared towards scrub typhus.

The purpose of this review is to provide an overview of the large historical impact of *O. tsutsugamushi*, with particular relevance to military operations; to describe the recent outbreaks of scrub typhus both within and beyond the Tsutsugamushi Triangle; to detail the information currently associated with antibiotic resistance to *O. tsutsugamushi*; and to summarize the current status of *Orientia* spp. genomics. It is our belief that a strong understanding of the past history, as well as, the present state of *Orientia* research must be considered when implementing successful future research strategies concerning *Orientia* spp. and related organisms. 

## 2. History and Military Relevance of Scrub Typhus

As a result of their dramatic impact on the military history of the world, the rickettsial diseases have significantly shaped the course of civilization. With the exception of malaria, no other class of infectious diseases has caused more suffering or loss of life. In his most famous work ‘Rats, Lice and History’, Hans Zinsser (1878–1940), the preeminent American physician and bacteriologist (Brill-Zinsser disease), wrote of an early account found in Thucydides’ History [81]. The text describes the louse-borne epidemic typhus as the purported cause of the plague of Athens, killing the Athenian general Pericles, and responsible for the defeat of Athens during the Peloponnesian Wars, about 429 B.C. Another example of the impact of rickettsioses, one of many, would be the disastrous retreat from Russia in the summer of 1812 by Napoleon’s Grande Armeé, when an estimated 100,000 soldiers died, primarily of typhus. During their retreat, ahead of the attacking Russian army, surviving French soldiers carried the infected lice back to western Europe, thus spreading the dreaded disease. During ‘The Great War’, or World War I, epidemic typhus was indeed epidemic, especially in eastern Europe, responsible for killing millions of civilians and soldiers [34]. Its impact was far less on the western front, probably due to the introduction of effective delousing efforts. These brief examples underscore a history replete with examples and descriptions of the strategic impact of epidemic typhus, especially on military operations. However, unlike the role played by epidemic typhus during World War I, the effect of another major rickettsiosis—scrub, or mite-borne, typhus—on military operations is more circumspect and focal, coming into its own in the next great war.

In a world on the brink of World War II, it became evident within the American military medical community that certain infectious diseases, including epidemic typhus and the tropical diseases, would likely have a major impact on the Allies. In anticipation of a need for medical officers trained in tropical disease diagnosis and treatment, a series of courses focused on the tropical diseases was designed and conducted at the Army Medical Department Research and Graduate School in Washington, DC, USA. Their effectiveness is well known and these courses are still being conducted annually at the Uniformed Services University of the Health Sciences, in Bethesda, MD, USA. Importantly, due to the proven wartime impact of epidemic typhus during World War I and over the centuries, outbreaks were anticipated and preparations to identify and control the disease were begun. Early in 1942, by presidential executive order, the joint (US Army-Navy-Public Health Service) United States of America Typhus Commission (Typhus Commission) was constituted and played an important role in wartime control of the rickettsioses. As expected, outbreaks of epidemic typhus were well-documented both during and immediately after the war in Naples, Italy; Cairo, Egypt; Hamburg, Germany; Iran; Morocco; Algeria; Japan; and Korea [82]. But because of effective treatment, primarily where civilian outbreaks occurred, including delousing with powdered DDT, few cases were reported in western Allied military personnel. Philip reports only 64 cases in the American Army during the entire war [83]. The very low morbidity and mortality was unexpected. However, the other typhus, scrub or mite-borne typhus, proved to be far more deadly to deployed military personnel. 

At the beginning of the war in the Pacific, the impact of scrub typhus on Allied forces had been totally unexpected. Still, scrub typhus had been recognized and studied by British scientists and physicians, as well as the Japanese, for some time [84]. In fact, this disease had been known locally for centuries [85]. The Japanese had identified what was likely scrub typhus in Niiagata Prefecture, Japan, and a description was published in 1878 by Baelz and Kawakami, who called the disease Japanese ‘blood fever’ or ‘river fever’ [85]. There had been reports both within Japan and in other regions within what had become to be called the Tsutsugamushi Triangle, including Sumatra (1902), the Philippines (1908), Queensland, Australia (1910), Vietnam, and Malaya (1915). Kawamura referred to a Chinese medical book in the 6th century describing a ‘sand louse’ vector found along river banks. In the 1930s, British scientists working at the Malayan Institute for Medical Research (IMR) in Kuala Lumpur were able to definitively separate typhus-like fevers into distinct entities: the urban form, known as urban or murine typhus; and rural typhus or scrub typhus [86]. Also during that era, the Weil-Felix OX-K test was developed to identify scrub typhus cases.

The Army Ground Forces Directive, dated 9 November 1944, notes that ‘Scrub typhus fever … in some operations … has disabled more men than has enemy action’ [87]. It was reported that, in some areas, it was second only to malaria in producing medical causalities in military personnel [83]. Impacts on Allied troops, i.e., United States, British, and Chinese troops, as well as the Japanese Army, proved far greater than typhus fever. As reported to the Office of the Surgeon General in a post-war summary, the number of scrub typhus cases in U.S. Army Forces from March 1942 through December 1945 was estimated at 6717 [83]. Altogether, there were approximately 18,000 scrub typhus cases reported among allied servicemen from 1941 to 1945 [88]. Although well known in Japan and to British scientists in the region, to U.S. military doctors, scrub typhus was initially considered to be of minor military importance. Described as ‘scrub itch’, the epidemiology of the disease was all but unknown. Attitudes soon changed as troops became ill or died of the disease in the Asia-Pacific theater of operations, initially in New Guinea and later in the China-Burma-India or CBI theater of operations, severely compromising combat capabilities [86]. In late summer 1942, scrub typhus cases were initially experienced in Australian forces, and subsequently in U.S. forces near Port Moresby, New Guinea [84]. Because of the outbreak, in October of 1943, field and laboratory studies were initiated under the direction of the Typhus Commission. In addition, the U.S. Navy deployed a medical research team to the region (NAMRU-2). Between 1942–1943, 957 cases and 53 deaths were recorded in Allied troops; and in one outbreak (Finschaven), a 35.3% fatality rate was recorded [86]. In December of 1944, another major outbreak was reported, this time in U.S., British and Chinese Forces in the CBI theater of operations. A second Typhus Commission group established a laboratory near the Stilwell Road, on the Assam-Burma border. In that region between 1 November 1943 and 1 September 1945, 1098 cases of scrub typhus and an 8.9% mortality rate were reported in United States and Chinese troops [86]. From August through November of 1944, British Army scrub typhus casualties were greater than those among the Americans, especially along the region known as the ‘Imphal’ [83]. Interestingly, captured medical documents described similar experiences in Japanese troops. However, they had failed to recognize that scrub typhus was Tsutsugamushi disease—the same as experienced and researched in their homeland. Clearly, scrub typhus had a major impact on military personnel on both sides.

Following the war, combat operations ceased, so few cases were reported. Only 8 cases of scrub typhus were diagnosed in United States military or United Nations peace-keepers during the Korean conflict [34]. However, in recent years, scrub typhus has remained a major cause of febrile illness in the Korean civilian community, especially in agrarian communities. In contrast to the Korean conflict, during the Vietnam conflict, military were greatly affected by scrub typhus, even when appropriate antibiotic therapy was readily available. The Armed Forces Epidemiology Board (AFEB) reported it to be a major cause of fevers of unknown origin (FUO) in U.S. troops. They reported that once malaria was excluded, scrub typhus was responsible for 20–30 percent the FUOs during that conflict [34,89].

Peace-time outbreaks of scrub typhus seem to recur periodically, as described in U.S. Marines training near Mount Fuji, Japan. The reappearance of scrub typhus, first described in a Japanese soldier in 1934, occurred in 1948 at Camp Fuji in U.S. troops training there [34]. Outbreaks recurred in 1953, 1959, and periodically both in U.S. Marines training there and in Japanese Ground Defense Forces. In recent years, two scrub typhus outbreaks occurred between 25 October and 3 November 2000, and 17 October and 30 November 2001. When analyzed in conjunction with the antibiotic-resistant scrub typhus reported in Thailand in 1995, outbreaks, such as those in Japan, remain a concern for deployed military [74,90]. 

Woodward describes the ‘disturbing enigma’ of mission-compromising diseases experienced in the Asia-Pacific war [88]. Neither preventive vaccines nor specific treatments, such as antibiotics, were available to control diseases such as malaria and scrub typhus, which had infected or killed so many. Although scrub typhus attack rates diminished near the war’s end, as Typhus Commission-driven mite-control studies and measures were developed and applied, a determined look forward would be needed [88]. Soon after the war, one highly successful world-class cooperative venture was undertaken. Late in 1947, a collaboration was arranged between the Malayan IMR (Dr. Raymond Lewthwaite, Director), and a U.S. Army team of doctors led by Dr. Joseph Smadel of the Army Medical Department Research and Graduate School (later renamed the Walter Reed Army Institute of Research or WRAIR). As the scientific director, Dr. Smadel had established an arrangement with Parke Davis and Company, Detroit, MI, USA, which permitted his team to test the newly developed antibiotics including their latest, chloromycetin, in scrub typhus patients. Since the just-completed in vitro and in vivo animal studies involving the orientiae using the new antibiotic had appeared quite promising, Dr. Smadel quickly arranged for testing in patients within the endemic area. Dr. Lewthwaite had identified to Smadel an area highly endemic for scrub typhus. Within hours of the team’s arrival in Kuala Lumpur, the new drug had been administered to a young Malayan soldier. Within 24 h the Weil-Felix OX-K positive, isolate-positive soldier became afebrile. This early success appeared promising, and testing was continued in a cohort of scrub typhus patients. Disease resolution in several more proven cases quickly followed. For the first time in human history, an antibiotic had cured scrub typhus. 

This historical accomplishment, and the overall success of the Typhus Commission and its associated laboratories and environmental teams during the war, led to the establishment of a series of other U.S. Army and U.S. Navy overseas research laboratories. This included ‘standing up’ the U.S. Army Medical Research Unit-Malaysia (USAMRU-Malaysia) on 12 June 1953, under an agreement between the AFEB and the British government. Until its closure in 1989, this unit conducted research on several infectious diseases, but especially scrub typhus. Scientists and staff were involved with all aspects of this disease, conducting clinical treatment and prophylaxis trials with newly developed antibiotics, and vector studies that included the establishment and characterization of the first *Orientia*-infected *Leptotrombidium* vector colony. Other laboratories involved in scrub typhus research included the Thai Army and U.S. Army components of the Armed Forces Research Institute of Medical Sciences (AFRIMS), in Bangkok. The AFRIMS was instrumental in identifying the index antibiotic-resistant scrub typhus cases [78]. It continues to maintain and perform vector studies using its unique *Orientia*-infected trombiculid mite colony, a necessary component in the testing of candidate scrub typhus vaccines. Navy laboratories overseas included the Naval Medical Research Unit No. 2 (NAMRU-2), initially located at Rockefeller University in 1944, then moved the next year to Guam to support WWII infectious disease research. Later in 1955 NAMRU-2 was re-established in Taipei, Taiwan, then later moved to Manila, Jakarta, and is now in Phnom Penh, Cambodia. The NAMRU-3 laboratory in Cairo and NAMRU-5 in Ethiopia were heavily involved in epidemic typhus studies. These past and existing laboratories have played important roles in all aspects of rickettsial research, including identification, treatment, and vector and reservoir studies. They were responsible during World War II for isolation of the prototypical scrub typhus (*O. tsutsugamushi)* strains, which in subsequent years have been essential in developing experimental vaccines, antibiotic drug studies, rapid diagnostic tests and epidemiologic investigations monitoring infectious disease incidence in regions considered militarily significant. 

On the home front, scrub and epidemic typhus research has been based in the Washington, D.C. area. These medical research institutions include the WRAIR, which is home to the Department of Rickettsial Diseases. Their cutting-edge scrub typhus work included the first cloning and DNA sequencing of an *O. tsutsugamushi* gene. The Naval Medical Research Institute (NMRI), Bethesda, MD, USA had traditionally focused on epidemic typhus research, but is now known as the Naval Medical Research Center (NMRC), and is very active in scrub typhus research, including holding patents on recombinant scrub typhus proteins and developing state-of-the-art diagnostics, such as a quantitative real-time PCR (qPCR) assay needed for vaccine evaluations [91]. In 1989, the rickettsial disease programs of these two institutions consolidated into the Joint Army-Navy Rickettsial Diseases Research Program, now located at the NMRC in Silver Spring, MD, USA [34]. The consolidated program works in synergy with the overseas research laboratories, providing collaboration expertise and guidance as needed to help lessen the impact of the rickettsioses on military missions. 

The impact of scrub typhus, a once-devastating military mission-impairing disease, appears to be mostly controlled using current treatments. As new, safer antibiotics come on line, they must be evaluated. Antibiotic efficacy will be a cause of concern, especially as antibiotic resistance is further proven or becomes more widespread. If this is so, an effective licensed vaccine may be needed to prevent the recurrence of the mission-debilitating experiences of World War II. 

## 3. Scrub Typhus Outbreaks

According to the World Health Organization (WHO), a disease outbreak is the occurrence of cases of a disease in excess of what would normally be expected in a defined community, geographical area, or season. Under this definition, a single case of a communicable disease long absent from a population, a disease caused by an agent (for instance a bacterium or virus) not previously recognized in that community or area, or the emergence of a previously unknown disease may also constitute an outbreak. Scrub typhus has been endemic in the Asia-Pacific region, bounded by Japan in the east, Pakistan in the west, Russia in the north and Australia in the south [3]. It accounts for up to 19% of patients admitted to hospitals with undifferentiated febrile illnesses [92]. Although scrub typhus was thought to be confined geographically to the Asia-Pacific, and that only one species of *Orientia* (*Orientia tsutsugamushi*) was thought to exist, this opinion was amended with the recent description of an *Orientia* sp. associated with a scrub typhus-like illness in Chile [3], the discovery of the new species, *Candidatus* Orientia chuto, isolated from a patient who visited Dubai, United Arab Emirates (UAE) [2], and the increasing reports of suspected cases of scrub typhus in Africa [93,94].

Scrub typhus occurred in epidemics among troops in the Myanmar, Sri-Lanka, India and West Bengal during the Second World War [95]. During this pre-antibiotic era, mortality rates were as high as 40–45% [96]. This was followed by a marked decline in the number of reported cases in humans, possibly due to the use of tetracycline and chloramphenicol, and pesticides [80]. In some regions, scrub typhus disappeared completely for some time, only to resurface in an epidemic form. In the Maldives, it was known to be prevalent during World War II, and then it disappeared, only to reemerge in a 2002–2003 epidemic, causing several morbidities and fatalities [97]. We speculate that one (or a few) strain(s) of *O. tsutsugamushi* may have been circulating in the Maldives during the WWII era, and that the population in the Republic of Maldives was immune to the pristine genotype(s). In a recent study, mutations or the introduction of a new strain(s) may explain the reemergence of scrub typhus in an epidemic form. Analysis of *tsa56* gene encoding a major surface antigen responsible for antigenic variation revealed multiple intragenic recombination events and a high rate of mutation [98]. It has also been suggested that the antigenic heterogeneity of the *Orientia* species may be the reason for the frequent outbreaks and re-infections occurring in the endemic regions [99]. The resurgence in the number of cases diagnosed with scrub typhus may also be due to enhanced awareness, leading to increased surveillance, coupled with the improvement of the diagnostic tests [100]. Additionally, the widespread use of *Orientia-*resistant beta-lactam antimicrobials coincided with the increased incidences of scrub typhus [101]. In this review, we have documented human outbreaks of scrub typhus from 2007 to the present, 2017 (Table 1) [19,95,99,102,103,104,105,106,107,108,109,110,111,112,113,114,115,116,117,118,119,120]. 

The baseline level of scrub typhus in the endemic regions may not be known, and therefore, some of the scrub typhus outbreaks described herein may be sporadic, unrelated cases of the same disease, particularly for the hospital-based studies conducted prospectively [121] or retrospectively [114,116]. In these outbreaks, the patients presented with an undifferentiated febrile illness of varying severity with symptoms ranging from fever, headache, cough, rash and/or eschar, myalgia, lymphadenopathy [19,108], to more dramatic ones such as multiple organ failure [95,109], and death in some of the outbreaks [95,104,105,114,115,116]. In a review by Taylor et al., it was observed that the case fatality rate can be as high as 30–70% in untreated cases [96]. 

The outbreak investigations used clinical diagnosis coupled with laboratory findings of one or more of the serological diagnostic tests such as Weil-Felix (WF), rapid immunochromatographic test (RICT), immunofluorescence assays, molecular tools such as polymerase chain reaction (PCR), and occasionally, culture of *Orientia* sp. from the outbreak cases [102]. For some of the outbreak investigations, other causes of undifferentiated febrile illness, such as dengue, malaria, leptospirosis, and typhoid fevers [104], were ruled out. During some of the outbreaks, diagnosis was based on detection of antibodies (IgM) using commercial ELISA assay(s) coupled with presence of eschar [107], or exclusion of other causes of fever [115], or response to treatment [109]. The outbreaks affected people of all ages, but some were reported exclusively among pediatric patients [19,95,104,108]. All outbreak cases that met the clinical and laboratory definition were treated using doxycycline, chloramphenicol or azithromycin (Table 1). For uncomplicated cases, and in older children, doxycycline was prescribed. Otherwise, in pediatric patients less than 8 years and pregnant women without complications, azithromycin was substituted for doxycycline [95,108,110,111]. In complicated scrub typhus cases, such as those with meningoencephalitis, septic shock, myocarditis, and/or multi-organ system involvement, broad spectrum antibiotics were indicated [95,108]. Case fatalities were most likely due to delayed diagnosis and treatment resulting in multi-organ system failure, as observed in some of the studies [104,109,112,115], or may be as a result of complications due to mixed infections (co-morbidities) observed in some of the outbreaks [109].

Factors that may have resulted in increased risk were identified in some of the outbreaks, including patients squatting when relieving themselves in the bushes [117]. In another outbreak, visiting Xiaogang Park in Guangzhou City, Guangdong, China, was indicated as the risk factor for the outbreak involving 29 patients reported to occur from 1 May to 17 June 2012 [105]. Bundling of waste straw was identified as a major risk factor for the scrub typhus outbreak that occurred in Jingjiang City, in south-central Jiangsu, China, from 18 October through 11 December 2013, with a peak in early to mid-November 2013, involving 272 cases [103]. In a recent outbreak that occurred in Nepal, involving 23 cases, large-scale destruction of human habitation, rendering people homeless, was identified as the risk factor [119]. In Australia, the most recent outbreak occurred in April 2011, among 124 infantry soldiers and support elements deployed to the Cowley Beach Training Area in northern Queensland, Australia [102]. The cases were determined by demonstration of a fourfold rise in IgG antibody titer to *O. tsutsugamushi* by serology, a positive by PCR, or obtaining a positive culture from clinical specimens. Previous outbreaks have occurred in the same training area among soldiers on military training exercises [122,123]. Some habitats, such as sandy beaches, mountain deserts, forest clearings, riverbanks, and grassy regions provide optimal conditions for infected mites to thrive [124]. In 2014, an outbreak involving nine scrub typhus laboratory-confirmed cases was reported in the western province of the Solomon Islands, located in the South Pacific region [120]. In this outbreak, both cases and controls reported presence of rats in their houses and gardens. All cases were males and presented with an undifferentiated febrile illness.

**Figure 1:**
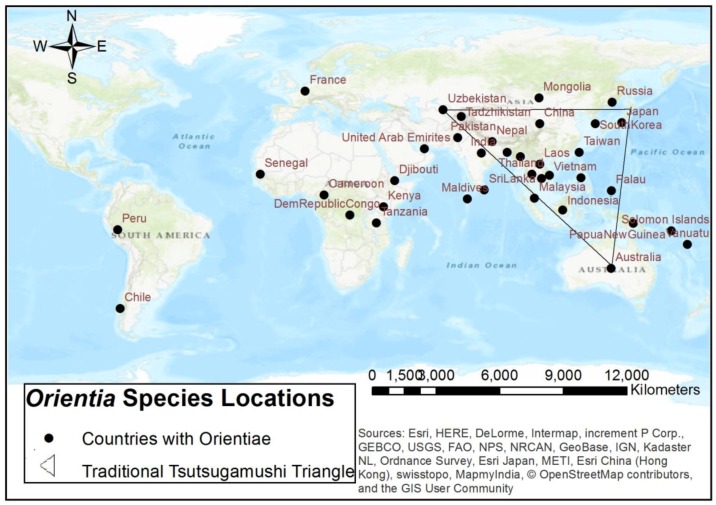
Distribution of *Orientia* species both within and beyond the endemic Tsutsugamushi Triangle.

In recent years, the use of GIS mapping to better understand disease outbreaks, distribution of associated vectors, and potential predictive factors has been employed for rickettsial and other vector-borne diseases [125,126,127,128,129]. In particular, the preliminary distribution of *Orientia* sp. has been described and visualized in several studies, reports, and reviews [23,81,100,130,131,132]. Traditionally, scrub typhus has been reported to be found within the endemic Tsutsugamushi Triangle of the Asia-Pacific geographic region and northern Australia [23,80,98,129,130,131,133]. Recent studies and reports of potential and confirmed cases, outbreaks, and serosurveys have indicated regions of emerging and new localities outside of the Tsutsugamushi Triangle that are not under surveillance for this agent and disease [2,3,30,94,130] (Figure 1). Kim et al. [98] suggested that the distribution that is being found to be emerging outside of the endemic triangle extends from the epicenter/focal point of Taiwan, which has a subtropical climate. Ecological, host, and human pattern changes may also factor into this analysis [23,98]. It has been proposed that studies to further understand the spatial distribution are needed and could also incorporate spatial modeling [129]. With additional epidemiological and spatial data and analysis, a better understanding of the different *Orientia* species and their potential geographic locations is possible.

## 4. Antibiotic Resistance

Until recently, clinical interest in scrub typhus has been minimal, as effective treatments have existed for many years, which when implemented in patients infected with this disease, rapidly result in improvement. The earliest treatment studies demonstrated chloramphenicol was effective in the treatment of scrub typhus cases [132,134]. Alternatively, studies demonstrated antimicrobial therapy with one of the tetracyclines, particularly doxycycline, was also an effective treatment choice for scrub typhus [135,136,137]. Although effective, the use of either chloramphenicol or tetracycline poses risks to certain populations (pregnant women and children). Adverse effects associated with both of these drug classes have been noted in these specific populations, and treatment with either of these drug classes during pregnancy or childhood should be avoided [136]. For this reason, alternative antimicrobial therapies have been examined for clinical use. More recently, additional antibiotic classes, such as macrolides, quinolones and rifampicin, have also proven to be effective choices in disease treatment and management, although with varying degrees of effectiveness [136,138]. However, in the 21st century, we have seen the emergence of a major health care issue evident across the globe: serious infections caused by bacteria that have become resistant to commonly-used antibiotics [139]. Previously reliable treatments for many bacterial agents have become ineffective with the increasing rate of antibiotic resistance, as well as the emergence of ‘superbugs’ (dangerous multidrug-resistant strains) [139], making this an area of increasing concern. Although the majority of currently reported scrub typhus cases occur in the Asia-Australia-Pacific region [133], historically, this disease has impacted military troops during times of conflict/war [34], and recent examples of scrub typhus impacting military personnel have occurred [34,133,140,141,142,143]. Endemic presence of this disease in certain areas of the world, coupled with the impact on military presence, (particularly in endemic areas), as well as the lack of an effective vaccine for scrub typhus, make this an increasing area of concern as reports of possible antibiotic-resistant cases in Thailand began to appear as early as the 1990s [90,144,145,146,147,148,149].

In 1996, Watt et al. reported that scrub typhus patients from Chiangrai (north Thailand) responded poorly to the recommended appropriate antibiotic treatment (200 mg doxycycline). Ninety per cent of the Thai patients responded (fever abated) to therapy within 48 h; however, only 1/3 of the patients with early, mild disease were free from fever after 48 h, and most had fever and other symptomology for 3 days. Infections from the western portion of Thailand responded more rapidly to treatment (median fever clearance time was 30 h) [90]. The median clearance time of the Chiangrai cases was 80 h, which is significantly longer than that observed in patients from the western region, and also longer than the clearance time found in the 128 patients reported from the series of cases from Vietnam [90]. The authors did not believe the Chiangrai cases could be explained by poor doxycycline absorption (checks of blood serum levels within 10 patients demonstrated adequate concentrations of the drug). However, three potential scenarios were hypothesized to explain the slow response within these particular patients, including: (1) the possibility of infection with more virulent strains of *O. tsutsugamushi*; (2) potentially from infection with resistant strains; or (3) a combination of the two [90]. Subsequently, evaluation of cell culture identified a single Chiangrai isolate (C3) that demonstrated doxycycline resistance [90]. Although this isolate demonstrated lowered virulence when the drug was absent (it infected fewer cells than the reference strain Karp), the isolate’s ability to invade was not affected by 4 μg/mL of doxycycline (bacteria which grow in the presence of this concentration are considered resistant). A second isolate (C27), was also found to demonstrate partial doxycycline resistance (a lower invasion rate was observed when compared to C3 (15% vs. 26% at 4 μg/mL, respectively); however, the invasion rate was still higher when compared to the reference strain Karp (15% vs. 2% at 4 μg/mL, respectively) [90]. These results were also observed in murine studies, where more deaths occurred in doxycycline-treated mice infected with Chiangrai isolates (67% survival, C3 and C27), as compared to mice infected with the prototype (Karp) strain (100% survival) [90]. The authors acknowledged two potential ways in which resistant strains might arise. The first, which is the more unlikely mechanism, involves dormant organisms within patients subjected to repeated or long-term exposure to antibiotics (some of the drug treatments are bacteriostatic and cases where long-term survival of *O. tsutsugamushi* in patients cured of symptomatic scrub typhus have been previously reported) [90,150]. However, the mechanism behind how a strain could be transmitted to another host is not known, as chiggers are the only known reservoir of infection, and there has never been a case of transmission from a scrub typhus patient to a chigger recorded [90,150]. A second mechanism explaining the development of resistance was proposed by the authors; this explanation involved drug-resistant strains developing in chiggers due to antibiotic supplementation of poultry feed in Thailand. Antibiotic uptake could potentially occur in mites as they feed on their rodent host, whose food source is often the grain intended for poultry. Since *O. tsutsugamushi* has been demonstrated to transmit transovarially, drug-resistant strains created in this manner could be passed on to the next generation of chiggers, and ultimately to a patient [90].

A second *O. tsutsugamushi* strain, also demonstrating reduced doxycycline susceptibility (designated AFSC-4), was isolated by the Royal Thai Army in 1990 from a human patient in Kanchanaburi (a province within western Thailand) [75]. This strain, although isolated in Thailand, was greater than 500 km from the Chiangrai isolated strains discussed previously. Previous information about this strain was not available; however, it was included in a 1995 study examining the in vitro effectiveness of azithromycin against *Orientia* strains, where it was described as a doxycycline-resistant isolate [74]. In this study, strain AFSC-4 required a doxycycline concentration of 0.25 mg/mL before a level of inhibition equivalent to that demonstrated by the control strain (Karp) was achieved. By examining the percentage of cells infected (as a measure of *O. tsutsugamushi* growth), the data suggested a 32-fold difference in MIC values when comparing doxycycline effectiveness in controlling infection by the two strains [74]. 

In a subsequent study conducted in 2000 (also in northern Thailand), Watt et al. examined the efficacy of using rifampicin in scrub typhus cases that were poorly responsive to standard antimicrobial therapy [149]. In this study, the authors indicated that rifampicin had been previously identified in mouse antibiotic susceptibility testing as significantly more active against three northern Thai strains of *O. tsutsugamushi* (as compared to doxycycline) [149]. The purpose of this 3-year study published in 2000 (conducted in the Chiangrai Regional Hospital) was to compare rifampicin with standard doxycycline treatment in potentially drug-resistant scrub typhus infections. This study demonstrated that scrub typhus infections were less susceptible to doxycycline as compared to rifampicin (15 out of 28 patients continued to demonstrate febrile symptoms 48 h after beginning treatment). For rifampicin, fever clearance time was approximately half that observed in doxycycline-treated patients [149]. The authors also noted two patients who relapsed, both of whom received doxycycline treatment (no patient relapses were recorded with rifampicin therapy). The results of this study indicate rifampicin treatment to be a viable alternative for treatment of *O. tsutsugamushi* infections, where drug-resistant strains have been found.

Additional evidence supporting the potential development of drug-resistant *O. tsutsugamushi* strains outside of Thailand appeared in 1999 in a study conducted in Indochina [140]. This study performed targeted surveillance from June 1996 through April 1998 in 347 U.S. personnel (mostly military), deployed to various areas within the region: Laos (127), Vietnam (194), and Cambodia (26). The purpose of this screening activity was to provide a measure of risk relative to possible *O. tsutsugamushi* exposure. The results of the study demonstrated evidence of fourteen seroconversions to *O*. *tsutsugamushi* (seroconversion was defined by a negative pre-deployment sample and a positive post-deployment sample or a four-fold increase in titer). The seroconversion results translated into an overall scrub typhus incidence of 4.0% (3.9% for Laos, 4.6% for Vietnam) [140]. No seroconversion was found in individuals followed in this study in Cambodia. The importance of exposures of these individuals to *O. tsutsugamushi* becomes apparent when considering that these individuals receive 100 mg of doxycycline daily as prophylactic prevention for *Plasmodium falciparum* infection. Despite this prophylactic treatment, 3 of the 14 study participants who seroconverted (21%) reported clinical signs and symptoms characteristic of *O. tsutsugamushi* infection: the presence of maculopapular rashes (mainly on the arms and legs), low-grade fever, and joint pain [140]. In comparison, a smaller percentage of study participants who did not seroconvert (ranging from 6.9–7.2%) reported rashes, fever, and joint pain [140]. Daily prophylactic administration of doxycycline failed to prevent the breakthrough infections and suggested the potential emergence of doxycycline-resistant strains.

More recent reported cases of suspected resistance involving multiple treatment failures have appeared in the literature associated with scrub typhus-infected patients. Mathai, in a study published in 2003, reported one fatal scrub typhus case involving a patient treated with doxycycline therapy, indicating the possibility of resistance (however, the author also indicated that treatment delay may have played a role in this outcome) [146]. In 2014, Lee reported a single case of *O. tsutsugamushi* meningoencephalitis that occurred despite treatment with standard doxycycline therapy [145]. Progression of scrub typhus-infected patients to central nervous system involvement occurs in approximately 2–5% of cases, but progression is unusual in the presence of doxycycline therapy [145,151]. In the aforementioned case, the clinical course of the disease worsened steadily in the patient, despite treatment with doxycycline, but quickly improved when treatment was replaced with azithromycin [145]. The authors hypothesized that the poor response to doxycycline may have been associated with the reduced susceptibility of *O. tsutsugamushi* strains within this region to doxycycline, as had been shown in previous studies [90,145]. In a 2016 study conducted in northeastern Thailand [148], the case fatality rate observed was 6.3%, which was higher than that observed in a study conducted earlier in Thailand (2000–2003), which reported a case fatality rate of 2.3%. Additionally, in 66.7% of the fatal cases (6 out of 9 cases), patients developed severe disease within one week of contracting the illness. This rapid progression to severe disease contradicts the literature, which indicates complications generally appear within the second week [148,152]. The authors suspected that treatment failure in deceased cases resulted from doxycycline resistance, but did not perform scrub typhus culture or conduct antimicrobial resistance testing to confirm this hypothesis. 

In addition to the naturally-occurring incidents within infected scrub typhus patients demonstrating reduced treatment efficacy, strains of *O. tsutsugamushi* resistant to a major class of antibiotics (fluoroquinolones) have been demonstrated. Previous studies have shown resistance of other intracellular bacteria (such as *Bartonella* spp.) as well as *E. coli* to fluoroquinolones [153,154,155,156]. In these studies, resistance was associated with point mutations occurring within the quinolone resistance determining region (QRDR) of DNA gyrase (*gyrA*). In a study published in 2010, Tantibhedhyangkul demonstrated a similar process in *O. tsutsugamushi* strains [147]. The authors found that the QRDR region of *gyrA* of 18 Lao *O. tsutsugamushi* strains displayed the Ser83Leu mutation. Two other common mutations observed were Thr88Ser (19 isolates) and Ile89Leu (20 isolates), while one isolate demonstrated a Thr88Ala mutation. Additionally, the *O. tsutsugamushi* strain Kato was found to be resistant to ciprofloxacin, and examination of all available sequences for the *O*. *tsutsugamushi* QRDR domain of *gyrA* demonstrated a mutation at position 83 (which has been associated with resistance to fluoroquinolones in other obligate, intracellular groups such as *Ehrlichia* spp., *Bartonella* spp. and *Tropheryma whipplei*) [153,154]. The data presented in this study suggests that strains of *O. tsutsugamushi* may be naturally resistant to the fluoroquinolone class of antibiotics. The data also supports the results of several other studies, where treatment failure occurred in clinical cases of scrub typhus infection [157,158,159]. However, it should be noted that these studies are contradictory to reports of successful fluoroquinolone treatment in other scrub typhus cases [160,161,162]. 

Beyond the potential resistance mechanisms proposed by authors in the studies mentioned briefly above, there may be other genes/systems present within *Orientia* species strains that could contribute to the potential of development of the antibiotic resistance of this organism. Currently, a small amount of sequence data (whole genome) is available for *O. tsutsugamushi* isolates [163,164,165,166]. Mining available sequence data could result in the detection of specific genes known to play a role in resistance (including but not limited to efflux transporters or family efflux transporters, multidrug transport systems and enzymes (beta-lactamases and/or permeases)). Detection of those genes known to play a role in resistance mechanisms would enhance future research examining potential genomic components that may contribute to the development of resistant *O. tsutsugamushi* strains.

While the studies discussed above present evidence for the existence of strains of *O*. *tsutsugamushi* potentially resistant to conventional antibiotic therapy, widespread distribution and an extensive presence of antibiotic-resistant scrub typhus strains have not been demonstrated. These studies do demonstrate the need for additional research to definitively answer the important public health questions surrounding scrub typhus, especially considering the ever-expanding endemic regions battling this disease and the significant impact on military personnel stationed throughout the world. Elucidation of the exact methods of resistance will become important for assessing the impact the development of resistant strains might have on the treatment of scrub typhus infections, worldwide.

## 5. Genotypes and Genome Sequencing

*O. tsutsugamushi* isolates were historically classified by their antigenic similarity to the three prototype isolates, Karp, Kato, and Gilliam [167,168]. This initial classification was based on serology determined by complement fixation (CF) analysis and later incorporating indirect and direct immunofluorescence assays (IFA and DFA) [80,167,168,169,170]. Later, protein analyses suggested that the 56-kDa protein was a major antigenic determinant that exhibits high antigenic variability [171,172,173]. Naturally, with the advent of PCR and sequencing technology, the 56-kDa became the initial focus of cloning and sequencing efforts [174,175,176]. These initial sequence analyses in the early 1990s by Ohashi et al. and Stover et al. began with the prototype isolates, but quickly expanded to include other prominent isolates through both sequencing efforts and restriction length polymorphism analyses (RFLP) [174,175,176,177]. As more isolates were analyzed, it was quickly evident that the high degree of antigenic variation observed with *O. tsutsugamushi* could be seen in the sequence analyses [174,177]. The 56-kDa gene sequences are about 1550 base pairs long, and the protein product has been shown to vary in size from 516 to 540 amino acid residues [80]. In addition, the 56-kDa protein contains four hypervariable regions, indicating a high level of genetic diversity among isolates [80,174,177]. 

Initial genotype analyses employed the 56-kDa gene for restriction fragment length polymorphism (RFLP) analyses [177,178,179]. Prototype strain RFLP patterns were compared to patterns observed for other *O. tsutsugamushi* isolates collected across wide geographic areas. These analyses indicated a high degree of diversity in the 56-kDa gene sequence, but isolates were often still categorized as Karp-, Kato-, or Gilliam-like, with some researchers adding additional prototype strains based on location, such as the Kawasaki, Kuroki and Shimokohi isolates from Japan [177,178,179]. RFLP analyses are based on both full 56-kDa gene sequences and nested PCRs amplifying select variable regions [177,178,179,180]. These analyses showed a high degree of variability between the digestion patterns for isolates, and resulted in the ability to break isolates down into even smaller groupings than could be seen previously with serology [177,178,179]. Additionally, discrepancies between groupings based on RFLP patterns and serology were also observed, further indicating that there is a high degree of genetic diversity among *O. tsutsugamushi* isolates [178]. 

In the late 1990s and early 2000s, sequencing of the 56-kDa gene for genotyping and phylogenetic analysis gained popularity. As with serology and RFLP, genotyping was based on similarities to Karp, Kato, and Gilliam, along with the inclusion of additional Thai (TA763 and TA716), Japanese (Kawasaki, Shimokoshi, Kuroki, and Saitama), and Korean (Boryong) reference isolates as distinct genotypes/clades [181,182]. Genotyping efforts have largely employed the entire open reading frame for the 56-kDa gene; however, some groups have focused on sequencing variable domains I, II, III, and IV [180,181,182,183,184,185]. Much of this work focuses on geographic distribution of genotypes and comparing *O. tsutsugamushi* isolated from humans, rodents, and chiggers to known prototype and reference strains using identity and phylogenetic analyses [181,182,183,184,185,186,187,188,189,190]. Data from these analyses indicate that isolate similarities range from 100% identity to reference or prototype strains, to as low as 60% identity, with the majority of sequences ranging from 95–80% identical [181,182,183,184,185,186,187,188,189,190]. Additionally, phylogenetic and sequence similarity data indicate geographic differences in predominant genotypes. Several studies have concluded that the majority of *O. tsutsugamushi* isolates in Korea are most closely related to the Boryong genotype, whereas publications, including those by Blacksell et al. and Parola et al., conclude that the most common genotypes in Thailand and Laos are Karp and Gilliam, respectively [181,186,189,190]. Sequencing of the 56-kDa gene of Malaysian isolates has shown these isolates to be most similar to Gilliam and other Thai reference strains, while sequencing of Indian isolates indicates these isolates are most similar to the Kato genotype [185,191]. Kelly et al. (2009) found that Karp was the predominant genotype globally and proposed 9 distinct clades/genotypes based on 135 nearly complete open reading frames sequences for the 56-kDa sequences found in GenBank [80]. These genotypes include Karp, Saitama, Kuroki, TA763, Gilliam, Kawasaki, Japanese Gilliam, Kato, and Shimokoshi [80]. While many *O. tsutsugamushi* isolates can be categorized into distinct genotypes, many cannot, and represent unique genotypes [180,183,184,192]. Moreover, the use of the 56-kDa gene for phylogenetic analyses might not be the most useful surrogate for isolate relatedness, due to its interaction with the host. The 56-kDa protein has been found to facilitate host cell uptake and is likely under extreme selective pressure, causing a diversifying effect and increased rates of recombination that might not be seen in other regions of the *O. tsutsugamushi* genome [98,193,194]. That being said, 56-kDa genotyping is still the most widely used method for identifying and genotyping *O. tsutsugamushi* isolates.

In addition to single-gene genotyping, multi-locus sequence typing (MLST) analyses have been employed in an attempt to extrapolate better evolutionary relationships between isolates, as these relationships are unlikely to be seen with the more widely used 56-kDa genotype analyses [98,193,194]. ‘Housekeeping’ genes, or conserved genes, are chosen for these analyses in an attempt to reduce changes in phylogenetic structuring due to selective pressure and recombination [194,195,196]. Interestingly, these analyses were not undertaken when the genomes of *O. tsutsugamushi* Boryong and Ikeda were sequenced [163,166,194]. While MLST analyses are commonly used to classify other *Rickettsia* spp., only a handful of MLST studies have been published for *O. tsutsugamushi*, since the first genomes were only made publicly available in 2007 [194,195,196,197,198]. These few studies attempted to use genes under neutral selection, and were chosen based on genes employed in MLST analyses for other rickettsial agents, analyses of conserved regions from the two publicly available genomes, and/or shotgun sequencing of a reference strain (UT76) [194,195,196,197,198]. A single study by Nakayama et al. that employed 11 housekeeping genes (*atpD*, *clpX*, *dnaJ*, *dnaK*, *fabD*, *gyrB*, *icd*, *mdh*, *nrdA*, *sucD*, and *ubi*) indicated a correlation between virulence in mice and phylogenetic structuring of Japanese isolates [194]. However, this has yet to be repeated, and most other MLST studies focused on the genetic diversity of *O. tsutsugamushi* [194,195,196,197,198].

Additionally, MLST studies have largely focused on clinical isolates from Southeast Asia. Two studies by Sonthayanon et al. and Phetsouvanh et al. used *gspA*, *mdh*, *nrdf*, *nuoF*, *ppdK*, *sucB*, and *sucD* for MLST analyses of clinical isolates from Thailand and Laos, while studies by Duong et al. and Wongprompitak et al. chose *adk*, *lepB*, *lipB*, *secY*, *sodB*, and *sucA* for their typing of clinical isolates from Cambodia and isolates from Southeast Asia, respectively [195,196,197,198]. These studies indicate that MLST provides a much higher discrimination of strain types (ST) as compared to genotyping by the 56-kDa gene [197,198]. Additionally, results from 56-kDa gene-sequence analyses were not consistent with results from the MLST schemes [197,198]. These studies consistently found high numbers of strain types [195,196,197,198]. In one study, there was a unique strain type assigned to every single isolate studied [195]. In addition, a much higher degree of diversity is seen among the *O. tsutsugamushi* single genes chosen for MLST compared to the levels of divergence for these same genes in other rickettsial species [195,198]. Furthermore, the results of the MLST analyses described high rates of homologous recombination, which, until recently, was not thought to occur in *O. tustusgamushi* due to its intracellular nature and lifecycle of vertical transmission [195,196,197,198]. Interestingly, the analyses of the Lao and Thai isolates resulted in distinct geographic clustering, while Duong et al. and Wongprompitak et al. failed to see any geographic clustering in their analyses [195,196,197,198]. In addition, both Sonthayanon et al. and Phetsouvanh et al. found high rates of co-infection (2 or more *O. tsutsugamushi* isolates) of 25% and 8%, respectively, while co-infection was not seen in Cambodia [195,196,197]. Unfortunately, given the evidence of high rates of recombination events and the large numbers of STs generated in each of the studies, these MLST schemes are not likely to provide a true representation of the *O. tsutsugamushi* phylogeny.

To date, only two fully assembled genomes, belonging to the Boryong (Korea) and Ikeda (Japan) isolates, exist for *O. tsutsugamushi* [163,166]. The genomes for these isolates were published in 2007 (Boryong) and 2008 (Ikeda) and, while no other fully assembled genomes have been made available in the last decade, the three prototype isolates, 6 additional reference isolates, and *Candidatus* O. chuto draft genomes are available through NCBI [163,166]. Additionally, 33 genomes (32 unique isolates) are available through NCBI as raw sequence data. Analyses of the two full Boryong and Ikeda genomes indicate a genome size of about 2.0–2.3 Mb, but found that over a third of the *O. tsutsugamushi* genome is composed of duplicated genes, with the Boryong and Ikeda genomes containing ~37 and ~46 duplicated genes, respectively [163,166]. These duplications include conjugative and transposable elements giving *O. tsutsugamushi* the most highly repetitive genome of any bacterial species [163,166]. The comparisons of the two genomes indicates that the large number of repeats seen in both genomes due to amplification of mobile elements has resulted in intense genome shuffling [163,166]. 

A recent analysis of a Karp draft genome indicates a genome size similar to that of Ikeda at approximately 2.0 Mb [164]. Unpublished analyses of the 2 full genomes, 5 draft genomes, and the raw sequence data for 33 genomes confirmed high levels of gene duplication. Phyolgenetic and pan- genome analyses of this data set also found extensive recombination throughout the entire *O. tsutsugamushi* genome (Fleshman et al., unpublished). This data confirms results suggesting high levels of recombination from the MLST analyses [195,196,197,198].

## 6. Serological Methods for Diagnosing Scrub Typhus

The presence of specific antibodies (IgM and IgG) against scrub typhus group orientiae (STGO) is evidence of a current or past infection. Immunoassays have been developed to detect those specific antibodies using whole cell or recombinant antigens [129]. A single sample with positive IgM antibodies has been associated with an acute/primary infection, while the presence of IgG antibodies does not differentiate a recent infection from a past infection, due to their long-term persistence in the blood; the presence of IgG antibodies is useful in determining the prevalence of scrub typhus in a particular population. Seroconversion or a 4-fold rise in IgG titer using paired serum samples (acute and convalescent) better support the diagnosis of scrub typhus.

Early serological assays, including the Weil–Felix and the complement fixation (CF) tests, will not be reviewed here due to the disadvantages of these assays both in sensitivity and specificity [129]. The IFA was developed in 1963 in an indirect IFA format using Karp and Gilliam strains for diagnosis of scrub typhus [199]. The ability of the assay in typing of scrub typhus using Karp, Kato and Gilliam strains with fluorescent antibody in a direct IFA format was subsequently demonstrated [200]. Karp, Kato, Gilliam, Litchfield, the Japanese isolates (Irie, Hirano, Shimokoshi, Kawasaki, Kuroki, 432H-2, and Yamazaki), Thai isolates (TC586, TA678, TA686, TA716, TA763, and TH1817) and Boryong strains of *O. tsutsugamushi* have been used alone or in various combinations as antigens with in-house developed IFA assays and/or commercial kits [201]. The identification of group-specific IgM antibodies to the various strains of *O. tsutsugamushi* provides strong evidence of recent active infection. The sensitivity and specificity of IFA with paired sera has been reported to be 91% and 96% for IgG and 85% and 98% for IgM, respectively, using a 1:400 cutoff [202].

Microimmunofluorescence assay (micro-IFA) adapted IFA to a micro-scale format [203], and has recently been commercially produced by Fuller Laboratory (Fullerton, CA, USA) [204]. The benefit of this new format is that it allows for more than one antigen to be put in a single well of a slide, with each antigen forming a micro dot that is separated from the others, granting the detection of antibodies in the serum samples against all individual antigens simultaneously. Although the IFA has served as a diagnostic gold standard method in scrub typhus serological detection for decades, the lack of standardization and the use of variable cutoff titers has shown lab to lab variability in reporting results [201]. Moreover, the majority of sero-epidemiology studies used a single cutoff titer to determine positive results, with a wide range of reported cutoff values from 1:10 to 1:400, which also led to subjective endpoints that caused incomparable results [201]. In addition, the high cost and the requirement of experienced personal to read the IFA slides have limited its use in resource-restricted areas. Due to these limitations, other serological tests have been used more often in scrub typhus diagnosis and surveillance, and the IFA has now been relegated to serve as a confirmation test [31,205]. 

The indirect immunoperoxidase (IIP) assay is similar to the IFA, but utilizes peroxidase-labeled antibody and a light microscope in lieu of the fluorescent-labeled antibody and fluorescence microscope. The assay was first reported in Japan in 1982, and the results in quantifying both IgG and IgM antibodies against STGO from patients using this new technique correlated well with IFA [206]. This assay has been used as a standard for evaluation of other immunoassays [202,207]. 

The enzyme-linked immunosorbent assay (ELISA) was developed in the late 1970s, and has served as an alternative serologic assay that can detect group-specific IgM and IgG [208]. Purified whole-cell preparations of STGO, mostly Karp, Kato and Gilliam strains or a mixture thereof, have been used as ELISA antigens [31,141]. The sensitivity and specificity from a study on Thai patients were 97% and 89% for IgG (1:1600 cutoff) and 94% and 91% for IgM (1:400 cutoff) [202]. In recent years, ELISAs have used recombinant proteins. These recombinant antigens include the 56-, 47- and 22-kDa proteins, as well as surface cell antigens (ScaA and ScaC) derived from different strains of *O. tsutsugamushi* [209,210,211,212]. The sensitivity and specificity of the ELISAs using recombinant antigens increased to 98% in IgM and IgG detection when the mixture of these recombinant antigens was used [211]. Some of the most attractive features of ELISAs are their capacity for high throughput, as well as their low cost, easy procedure, reproducibility of results, and objectivity in performing the assay. These qualities are also particularly useful for surveillance studies [213,214]. Additionally, the ELISA using whole-cell antigens of Karp, Kato and Gilliam strains of *O. tsutsugamushi* has been shown to be useful for detecting antibodies against new species/strains of *Orientia* outside of the traditional Tsutsugamushi Triangle [2,3,31,205]. 

Immunochromatographic rapid diagnostic tests (RDT or ICT) have been developed in the last decade following the need of rapid diagnostic tools for the diagnosis of scrub typhus. The ability to produce high-quality recombinant protein antigens has made it possible to develop specific and sensitive RDTs. A mixture of three recombinant 56-kDa antigens from Karp, Kato, and Gilliam strains of *O. tsutsugamushi* was recently used to produce the Scrub Typhus CareStart tests manufactured by AccessBio (Somerset, NJ, USA). The lateral-flow-format showed good sensitivity and specificity for the detection of IgM (96.8% and 93.3%), but lower specificity for detecting total antibodies (71.4%), though the sensitivity was high (97.6%) [215]. More recently, a second-generation lateral-flow-format product, ScrubTyphus Detect (ST Detect), manufactured by InBios (Seattle, WA, USA) using a mixture of 4 recombinant 56-kDa antigens from Karp, Kato, Gilliam and TA763 strains of *O. tsutsugamushi*, showed improved sensitivity for IgM detection of 99.25 and 100% among Indian and Thai patients, respectively [216,217]. RDTs produce rapid results and follow a simple protocol with no need for sophisticated electrical equipment; they meet the requirements of a field-deployable, point-of-care diagnostic assay for the early diagnosis of scrub typhus with military relevance, and are also attractive for use in rural areas where the use of diagnostics like ELISA and IFA may not be available.

## 7. Conclusions

Scrub typhus and rickettsial diseases have long plagued both civilian and military populations throughout the world. Currently, scrub typhus outbreaks are being reported both within the known area of endemicity, as well as beyond the originally defined borders of the Tsutsugamushi Triangle. More concerning is the recognition of diseases caused by *O. tsutsugamushi* and other *Orientia* species far beyond the endemic region (Middle East, Africa, South America). Adding to this concern is the emergence of antibiotic-resistant strains of *Orientia* species, which can make treatment difficult or unsuccessful, even in the most well-equipped medical/laboratory facilities. Additionally, the ’flu-like nature associated with the scrub typhus group of diseases is another confounding factor that can delay diagnoses and/or lead to misdiagnoses, which can ultimately result in complications for the patient. As such, the continued pursuit of diagnostic tools, enhanced therapeutics, and ultimately a broadly protective vaccine, will all be key to the reduction of the global disease burden associated with *O. tsutsugamushi* and other *Orientia* species. Challenges still remain surrounding the laboratory maintenance/propagation of *O. tsutsugamushi* (need for BSL3 containment, skilled personnel, and specialized equipment) and the standardization of animal challenge models (related to mouse strain and challenge-route differences). However, researchers around the globe maintain the same goal of pursuing research geared towards better defining the pathogenesis of *O. tsutsugamushi* and its relation/translation to human scrub typhus cases. With the advent of whole genome sequencing and advanced genomic analyses, these techniques will undoubtedly be invaluable in the pursuit of vaccine candidates, the ultimate defining of molecular and immunological markers of pathogenesis, and the identification of novel genomic components that distinguish mild from severe strains of *Orientia* species. In conclusion, we have reviewed components of the historical, current, and potential future issues/events associated with *O. tsutsugamushi*, all of which are relevant to, and should be considered in, all facets of future scrub typhus research endeavors.

## Figures and Tables

**Table 1:** Human outbreaks of scrub typhus from 2007 to present, 2017.

Country	Province/State	Year	^#^ Laboratory Confirmed or Suspected Cases	Fatalities	Outbreak Duration	Age in Years	Treatment	Laboratory Diagnosis	Other Differentials Ruled Out	Reference
Australia	Northern Queensland	2011	124	0	21 April–5 May 2011	>18	Doxycycline	IFA, PCR, isolation	Not done	Harris et al., 2016
China	Jiangsu	2013	271	0						Hu et al., 2015
China	Guangdong	2012	29	4	1 May–17 June 2012	24–86	Not provided	WF, PCR,	Weil-Felix, PCR	Wei et al., 2014 ^#^
Bhutan	Thimphu	2014	12	2	August–October 2014	6–15	Not provided	RDT, IFA	Malaria, dengue, *S. enteritica*	Tshokey et al., 2017
India	Northern India	2013–2014	228	0	July 2013–31 December 2014	>12	Doxycycline, azithromycin, ceftriaxone	ELISA IgM, PCR & sequencing	Malaria, Widal, leptospirosis	Sharma et al., 2016
India	Himachal Pradesh (northern India)	2008	5	1	Not provided	15–Mar	Doxycycline, azithromycin ^$^ ceftriaxone ^^^	WF, MIF	Rickettsial only	Mahajan et al., 2008 ^#^
India	Pondicherry	2008	50	1	April 2006–April 2008	14–91	Doxycycline	WF, eschar	Widal, dengue, malaria leptospirosis	Vivekanandan et al., 2010
India	Meghalaya	2010	24	0	October 2009–January 2010	Pediatric	Doxycycline, Chloramphenicol ^^^ Azithromycin ^$^	WF	Malaria	Dass et al., 2011 ^#^
India	Meghalaya	2012	90	5	September 2011–August 2012	Adults	Doxycycline, azithromycin ^$^	Rapid immunochromato-graphic test	Typhoid fever, malaria, leptospirosis, dengue	Sivarajan et al., 2016
India	Puducherry	2013	28	0	September 2012–March 2013	1–89	Doxycycline, azithromycin ^$^	Rapid immunochromato-graphic test, ELISA, WF paired sera	Typhoid, dengue, leptospirosis, malaria	Stephen et al., 2015
India	Sikkim	2011	63	0	January–December 2011	>2	Doxycycline, azithromycin ^$^	Rapid immunochromato-graphic test, WF, ELISA,	Widal, Malaria, urine culture	Gurung et al., 2013
India	Rajasthan	2012	42	7	October–December 2012	3–78	Doxycycline	ELISA IgM	Widal, malaria, dengue	Sinha et al., 2014
India	Chennai	2011	52	0	September 2010–June 2011	0–16	-	ELISA, IgM, eschar	Not provided	Krishna et al., 2015
India	Rajasthan	2014	66	14	July–October 2014	Not provided	Doxycyline * azithromycin	ELISA IgM	Malaria, leptospirosis, dengue, typhoid, viral pharyngitis	Takhar et al., 2017
India	Uttarakhand	2014	69	1	July–November 2013	12–80	Not provided	IgM ELISA	Typhoid fever, malaria, dengue	Singh et al., 2014
India	Andhra-pradesh	2014	176	8	August 2011–December 2012	>12	Doxycycline	Weil-Felix, rapid immunochromato-graphic test	Other causes excluded	Subbalaxmi et al., 2014 ^#^
India	Andhra-pradesh	2011–2013	258	0	September–December 2011–2013	2–89	Tetracyclines	IgM ELISA, PCR	Typhoid, chikungunya, dengue	Usha et al., 2016
India	Bishnipur-Manipur	2007	38	2	May–September 2007	0–≥45	Doxycyline, azithromycin	WF	Not provided	Singh et al., 2010
Thailand	Chiang Mai	2007	65	0	June 2006–May 2007	0–13	Not provided	IFA, PCR isolation	Not provided	Rodkvamtook et al., 2013
Nepal	Chaitwan District	2016	264	8	May–October 2016	Not provided	Not provided	Not provided	Not available	http://www.myrepublica.com/news/7173/
Nepal	Kathmandu	2015	23	0	August–October 2015	Not provided	Doxycycline, azithromycin, ceftriaxone	ELISA IgM	Not available	Bastola and pant, 2016
Solomon Islands	Western Province	2014	9	0	1–30 May 2014	>11	Doxycycline, chloramphenicol ^$^	IFA/MIF	Malaria	Marks et al., 2016

* Other antibiotics administered before confirmation of diagnosis. ^$^ treatment in children younger children or pregnant women. ^#^ Retrospective study. ^^^ treatment in complicated scrub typhus cases.
